# High-fat diet attenuates the improvement of hypoxia-induced pulmonary hypertension in mice during reoxygenation

**DOI:** 10.1186/s12872-021-02143-x

**Published:** 2021-07-06

**Authors:** Koichi Sugimoto, Tetsuro Yokokawa, Tomofumi Misaka, Takashi Kaneshiro, Akiomi Yoshihisa, Kazuhiko Nakazato, Yasuchika Takeishi

**Affiliations:** 1 Department of Cardiovascular Medicine, Fukushima Medical University, Hikarigaoka 1, Fukushima, 960-1295 Japan; 2 Department of Pulmonary Hypertension, Fukushima Medical University, Hikarigaoka 1, Fukushima, 960-1295 Japan

**Keywords:** Pulmonary hypertension, Reverse remodeling, Obesity, Metabolic disorder, Hypoxia, Apoptosis

## Abstract

**Background:**

It is widely recognized that metabolic disorder is associated with pulmonary hypertension (PH). It is known that hypoxia-induced elevated pulmonary artery pressure in mice returns to normal pressure during reoxygenation. However, it is still unclear how metabolic disorder affects the reverse remodeling of pulmonary arteries. In this study, we investigated the effects of high-fat diet (HFD) on the decrease in pulmonary artery pressure and reverse remodeling of pulmonary arteries in mice with hypoxia-induced PH.

**Methods:**

We used female C57BL/6 mice aged 8 weeks. After being exposed to hypoxia (10% oxygen for four weeks) to induce PH, the mice were returned to normoxic conditions and randomized into a normal diet (ND) group and HFD group. Both groups were fed with their respective diets for 12 weeks.

**Results:**

The Fulton index and right ventricular systolic pressure measured by a micro-manometer catheter were significantly higher in the HFD group than in the ND group at 12 weeks after reoxygenation. The medial smooth muscle area was larger in the HFD group. Caspase-3 activity in the lung tissue of the HFD group was decreased, and the apoptosis of pulmonary smooth muscle cells was suppressed after reoxygenation. Moreover, the expression levels of peroxisome proliferator-activated receptor-γ and apelin were lower in the HFD group than in the ND group.

**Conclusions:**

The results suggest that metabolic disorder may suppress pulmonary artery reverse remodeling in mice with hypoxia-induced PH during reoxygenation.

**Supplementary Information:**

The online version contains supplementary material available at 10.1186/s12872-021-02143-x.

## Introduction

Hypoxia-induced pulmonary hypertension (PH) is a very prevalent form of PH in humans [1]. Hypoxia induces pulmonary vasoconstriction and pulmonary artery remodeling, which is characterized by organic stenosis due to abnormal proliferation of pulmonary artery smooth muscle cells [2, 3], whose mechanisms are not yet thoroughly understood.

It is known that hypoxia-induced elevated pulmonary arterial pressure in mice returns to normal pressure during reoxygenation [4, 5]. It is reported that increased pulmonary smooth muscle cell apoptosis and mitochondrial function were associated with pulmonary arterial reverse remodeling by reoxygenation [4, 6].

Several studies have reported that metabolic disorder is associated with PH [7, 8]. Peroxisome proliferator-activated receptor-γ (PPAR-γ), an important key molecule for metabolic syndrome, plays a crucial role in the pathogenesis of PH [9–11]. Alastalo et al. reported that PPAR-γ/β-catenin axis upregulates apelin that induces pulmonary smooth muscle cell apoptosis [11].

However, it is still unclear how metabolic disorder affects the reverse remodeling of pulmonary arteries. In this study, we investigated the effects of high-fat diet (HFD) on the decrease in pulmonary artery pressure and reverse remodeling of pulmonary arteries using mice with hypoxia-induced PH.

## Methods

### Animals and ethics statement

Littermate female C57BL/6 mice aged eight weeks (body weight range: 18.1–20.0 g) were used. The mice were housed with food and water ad libitum at room temperature at a 12:12 light-dark cycle. The investigations conformed to the Guidelines for the Care and Use of Laboratory Animals published by the US National Institutes of Health (NIH publication, 8th Edition, 2011). Our research protocol was approved by the Fukushima Medical University Animal Research Committee. The study on animals was carried out in compliance with the ARRIVE guidelines (Additional file 1). All efforts were made to minimize the suffering of animals, all of which were sacrificed by cervical dislocation after the experiments.

### Animal experiment

**Fig. 1:**
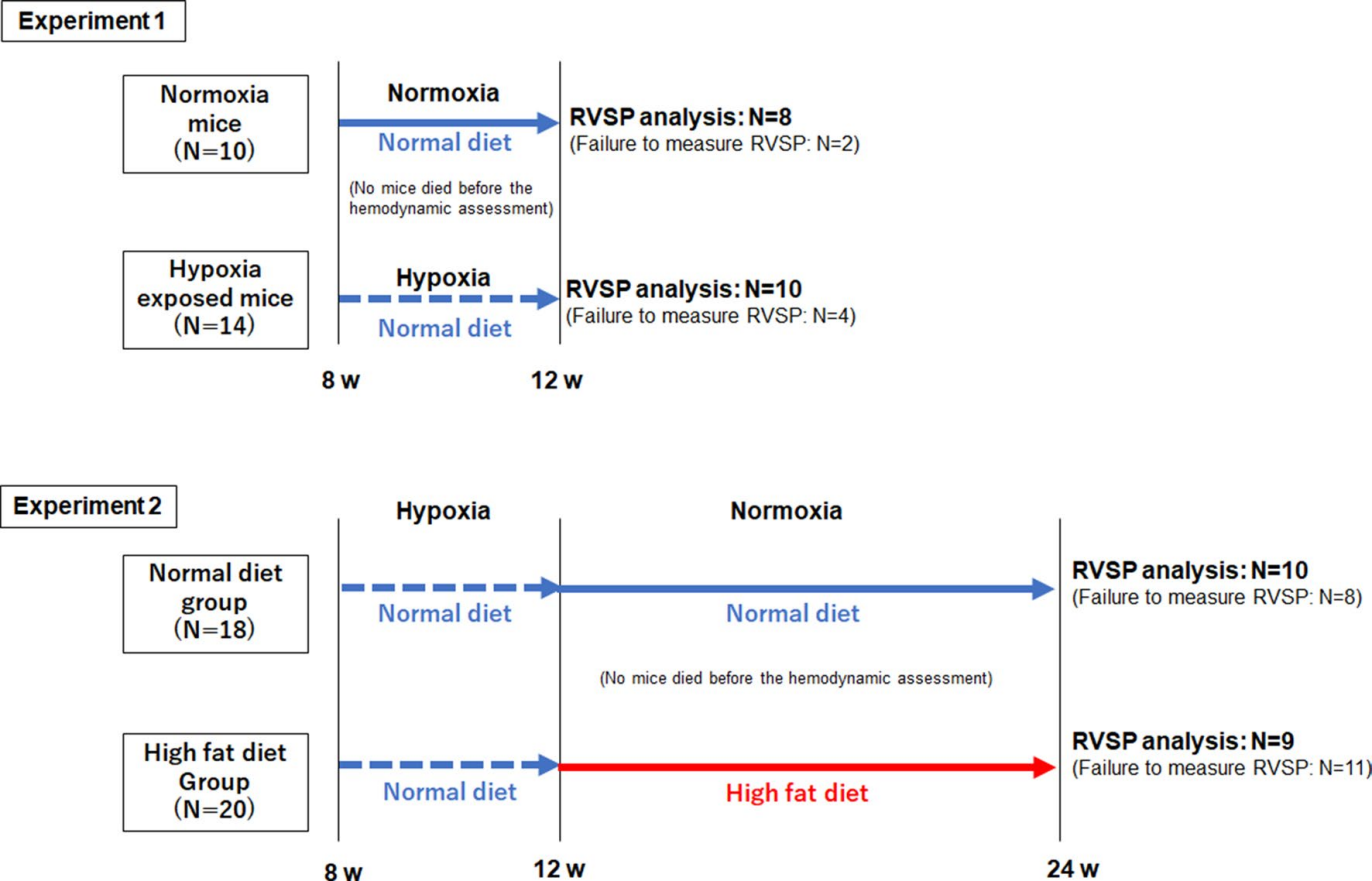
Experimental groups and protocols in mice. In experiment 1, the mice were exposed to normoxia or hypoxia with ND for 4 weeks. In experiment 2, the mice were exposed to hypoxic condition for four weeks and divided into ND and HFD group during reoxygenation (normoxia) for 12 weeks

The experimental protocol is shown in Fig. 1. Experiment 1: The mice were exposed to hypoxic (10% oxygen) or normoxic conditions for four weeks, during which they were given a normal chow. Thereafter, we evaluated the right ventricular systolic pressure (RVSP), right ventricular hypertrophy (RVH), expression of some associated molecules, and histological features. Experiment 2: The mice were exposed to hypoxic condition (10% oxygen) for four weeks, during which they were given a normal chow. Then, normoxia was resumed, and the mice were divided by a person, who was blinded to the investigation, into two groups; the ND group mice were fed with a normal diet (CLEA Rodent Diet CA-1, CLEA Japan Inc., Tokyo, Japan), while the HFD group mice were fed with HFD (High Fat diet 32, CLEA Japan Inc.) for 12 weeks (Table 1). The oxygen concentration of hypoxia was determined based on a previous study [4]. The required sample size was estimated to be 10–20 per group based on the number within the range reported in previous studies [12, 13]. The used animal number was described in each figure legend. The total number of animals used was 62. The mice in all groups were treated at once, and each group was allocated in each cage. After we unified the cage locations, we randomly measured the RVSP to avoid the effect of the order of animals and cages.

**Table 1:** Comparison of diet composition

	ND	HFD
*Nutrient components and calories*		
Moisture (%)	8.48	6.2
Crude protein (%)	27.09	25.5
Crude fat (%)	4.69	32.0
Crude fiber (%)	4.54	2.9
Crude ash (%)	7.92	4.0
Nitrogen free extracts (%)	47.2	29.4
Energy (kcal)	339.7	507.6
*Fatty acid composition (relative percentage to the total fatty acid)*		
Total (%)	100.0	100
Saturated fatty acid (%)	21.05	22.3
Monosaturated fatty acid (%)	28.47	66.5
Polyunsaturated fatty acid (%)	47.13	10.4
Myristic acid (%)	1.51	1.1
Myristoleic acid (%)	–	0.3
Pentadecanoic acid (%)	–	0.1
Palmitic acid (%)	16.71	1.2
Palmitoleic acid (%)	2.46	0.4
Heptadecanoic acid (%)	1.08	0.4
Heptadecenoic acid (%)	–	0.3
stearic acid (%)	2.45	7.5
oleic acid (%)	20.35	64.3
Elaidic acid (%)	0.18	–
Vaccenic acid (%)	3.29	–
Linoleic acid (%)	36.37	10.2
α-Linolenic acid (%)	3.08	0.2
Arachidic acid (%)	–	0.3
Icosenoic acid (%)	0.84	0.3
Arachidonic acid (%)	0.18	–
Eicosapentaenoic acid (%)	5.23	–
Behenic acid (%)	0.20	0.2
Docosahexaenoic acid (%)	2.27	–
Lignoceric acid (%)	0.18	–
Tetracosenoic acid (%)	0.27	–
Unidentified (%)	3.35	0.26

*ND* normal diet, *HFD* high-fat diet

### Measurements of right ventricular pressure and ventricular weight

Intraperitoneal injection of anesthesia was done using Tribromoethanol (0.25 mg/g of body weight). At 21% O_2_ condition, A 1.2-F micromanometer catheter (Transonic Scisense Inc., London, ON, Canada) was inserted to the right jugular vein, and the RVSP was measured without a ventilator and analyzed by LabScribe3 software (IWORX, Dover, NH, USA) [14]. Then, the mice were sacrificed by cervical dislocation; the heart and lungs were removed for RVH evaluation. The right ventricle (RV) was dissected from the left ventricle (LV), including the septum (S), and the RV/LV + S weight ratio (Fulton index) was calculated [14, 15]. The mice with severe bleeding or pneumothorax that affected the hemodynamics during catheterization were excluded from the RVSP analysis, as shown in Fig. 1.

### Histological analysis

After measuring the RV pressure, the right lung was fixed with 4% paraformaldehyde, embedded in paraffin, and sectioned to 3 μm. After Elastica-Masson (EM) staining or immunostaining of α-smooth muscle actin (α-SMA) (Santa Cruz Biotechnology Inc., Santa Cruz, CA, USA), the pulmonary arteries (external diameter of 20–50 μm) were randomly selected (60–90 vessels per individual mouse). The medial wall area (the area between the internal and external lamina) was measured using the Image J 1.48 software (National Institutes of Health, Bethesda, MD, USA) and divided by the vessel area (the area surrounded by the external lamina) [14, 15]. Furthermore, each vessel (external diameter of < 20 μm) was classified as non-muscular, partially muscular, or fully muscular, as described in a previous report. The percentage of muscularized pulmonary vessels was determined by dividing the sum of partially and fully muscular vessels by the total number of vessels [14]. Measurements were performed blinded to the mouse information.

### Lipids profiles

The plasma from mice at 24 weeks was isolated by centrifugation (3000 rpm, 15 min), frozen at − 80 °C, and analyzed by Nagahama Life Science Laboratory (Shiga, Japan). The plasma concentrations of high-density lipoprotein cholesterol (HDL-C) and low-density lipoprotein cholesterol (LDL-C) were measured by direct method using Chorestest N HDL® (Sekisui Medical Co., Ltd. Tokyo, Japan) and Chorestest LDL® (Sekisui Medical). The plasma triglyceride (TG) was measured by enzymatic method using L-type Wako TG∙M® (FUJIFILM Wako Pure Chemical Corporation. Osaka, Japan).

### Measurements of mitochondrial ATP

To evaluate the mitochondrial function, we measured the mitochondrial adenosine triphosphate (ATP) in the lungs based on a previous study [4]. The removed left lung was homogenized, and the mitochondrial fraction was isolated by centrifugation. Then, the ATP production was measured using a commercially available kit (TOYO B-Net Co., Ltd. Tokyo, Japan), following the manufacturer’s instruction.

### Mitochondrial DNA quantification

Mitochondrial DNA was quantified as described previously [16]. The DNA from the left lung was extracted and purified by QIAamp DNA Mini Kit (Qiagen, Hilden, Germany) according to the manufacturer’s instruction, with the inclusion of RNAse A digest. Quantitative polymerase chain reaction (PCR) was performed using the power SYBR green PCR master mix with a CFX Connect real-time PCR System (Bio-Rad Laboratories, Inc., Hercules, CA) using mitochondrial DNA-specific primer, cytochrome oxidase subunit 1 (Co1) gene, nuclear DNA-specific primer, and NADH:Ubiquinone oxidoreductase core subunit V1 (NDUFV1). The sequences of primers were as follows: forward 5′-TGCTAGCCGCAGGCATTAC-3′ and reverse 5′-GGGTGCCCAAAGAATCAGAAC-3′ for Co1, and forward 5′-CTTCCCCACTGGCCTCAAG-3′ and reverse 5′-CCAAAACCCAGTGATCCAGC-3′ for NDUFV1. All reactions were run in duplicates. The mitochondrial DNA content was calculated using the delta CT method, and the data were expressed as a fold increase in the ND group.

### Western blotting

The removed whole left lungs were used for western blotting, which was performed as described previously [17, 18]. Polyvinylidene difluoride membranes were incubated with mouse monoclonal antibodies to β-actin (Santa Cruz) and heme oxygenase-1 (HO-1) (Novus Biologicals USA, Littleton, CO, USA) diluted to 1:1000 for 1 h at room temperature. Rabbit polyclonal antibodies to active caspase-3 (Cell Signaling Technology, Beverly, MA, USA), PPAR-γ (Cell Signaling Technology), voltage-dependent anion channel (VDAC) (Cell Signaling Technology), hypoxia-inducible factor-1α (HIF-1α) (Cell Signaling Technology), and apelin (Bioss Inc, Boston, MA, USA) were diluted to 1:500. Then, each membrane was incubated with a horseradish peroxidase-conjugated secondary antibody or IRDye® 680RD or IRDye®800CW (LI-COR, Inc., Lincoln, NE, USA) diluted to 1:10,000 for 45 min.

### Apoptosis of pulmonary smooth muscle cells

To estimate the apoptosis of pulmonary smooth muscle cells, terminal deoxynucleotidyl transferase-mediated dUTP nick end-labeling (TUNEL) assay was performed by CF™640R TUNEL Assay Apoptosis Detection Kit (Biotium, Hayward, CA, USA), following the manufacturer’s instructions. α-SMA was detected by immunofluorescent staining using an antibody against α-SMA (Santa Cruz) and Alexa Fluor 488-conjugated goat anti-mouse secondary IgG (Abcam, Cambridge, United Kingdom). The samples were mounted with ProLong Gold Antifade Reagent with DAPI (Thermo Fisher Scientific, Waltham, MA, USA). Fluorescence was observed with an immunofluorescence microscope (BZ-X700, KEYENCE Co., Osaka, Japan). We randomly selected at least 10 fields in each specimen and counted the nuclei in the medial smooth muscle layer. The results were expressed as percentages of the number of TUNEL-positive nuclei over the total number of nuclei [14].

### Statistical analysis

The data are expressed as mean ± SEM, and statistical analysis was performed using either Student’s t-test or one-way ANOVA with Tukey’s multiple comparison test. A value of *P* < 0.05 was considered statistically significant.

## Results

### Effect of hypoxia on RVSP and RVH

**Fig. 2:**
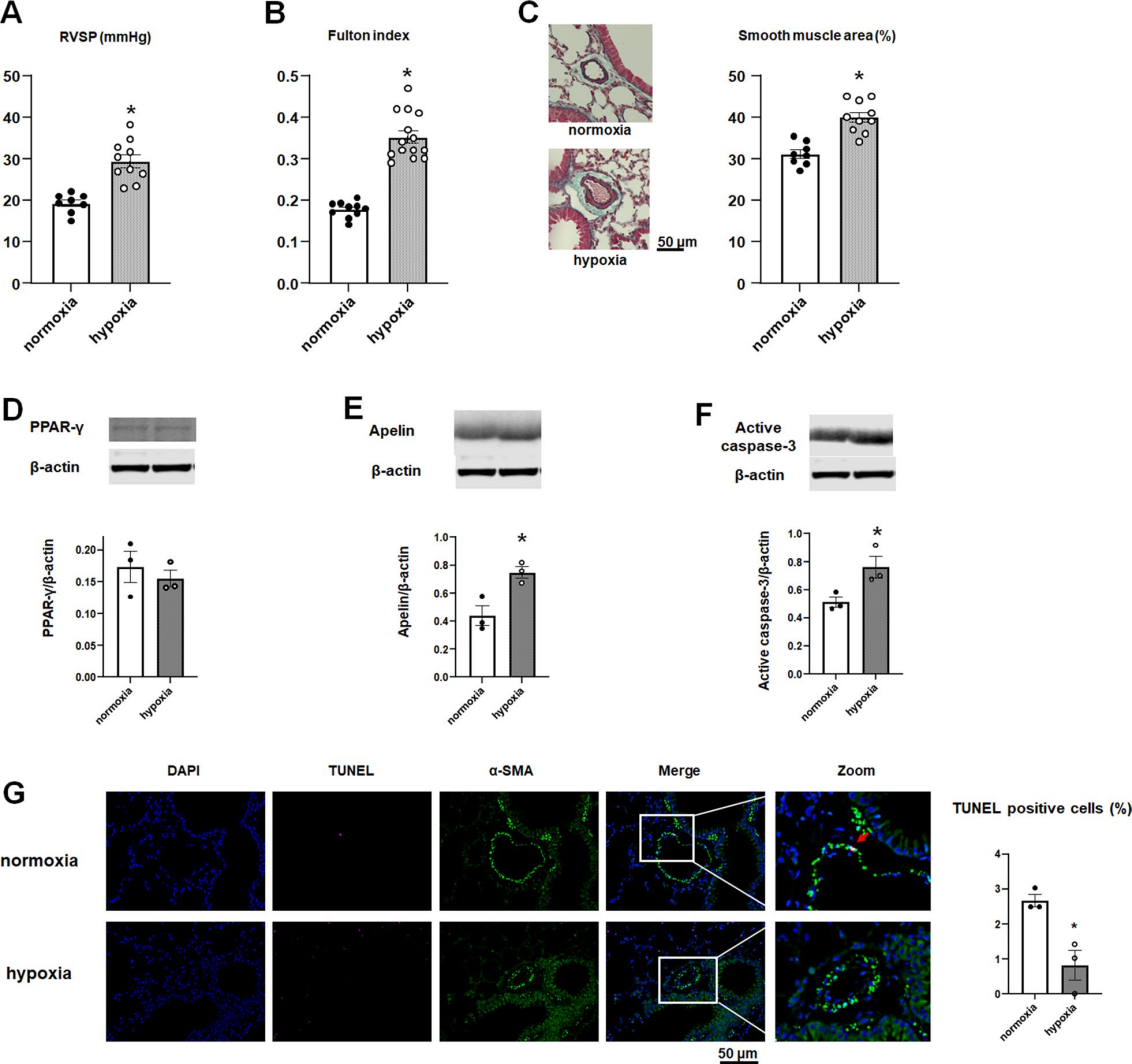
Effect of hypoxia on RVSP and RVH. Female C57BL/6 mice were exposed to normoxia or hypoxia for four weeks (Experiment 1). **A** The RVSP was measured by a micromanometer catheter. **B** Fulton index. **C** Representative microphotographs of pulmonary arteries after EM staining. The smooth muscle area was calculated and the results are expressed as mean ± SEM of 8 to 10 animals. *P < 0.05 versus normoxia group. **D**–**F** The expressions of PPAR- γ (**D**), apelin (**E**), and active caspase-3 (**F**) in the lung tissues determined by western blotting. Representative immunoblots (top panels) and bar graphs (bottom panel) are shown. The results are expressed as mean ± SEM (n = 3 in each group). *P < 0.05 versus normoxia group. **G** Representative microphotographs of the pulmonary arteries from normoxia and hypoxia group after TUNEL staining. TUNEL-positive cells are indicated by a red arrow. The graph represents the percentage of TUNEL-positive nuclei in the medial smooth muscle layer (n = 3 in each group)

In experiment 1, we confirmed the effect of hypoxia exposure on RVSP, RVH, and medial thickening at 12 weeks. Both RVSP and Fulton index are elevated in response to hypoxia (Fig. 2A, B). The medial smooth muscle layer of the hypoxia-exposed mice was significantly greater than that of the normoxia-exposed mice (Fig. 2C). Western blot demonstrated that PPAR-γ expression in the normoxia and hypoxia group is comparable, whereas the lung tissue apelin of hypoxia-exposed mice is higher than that of normoxia-exposed mice (Fig. 1D, E). The active caspase-3 in the whole lung tissue of hypoxia-exposed mice is increased. TUNEL staining showed that smooth muscle cell apoptosis is decreased in hypoxia-exposed mice (Fig. 1F, G).

### Effect of HFD on hypoxia-induced PH mice during reoxygenation

**Fig. 3:**
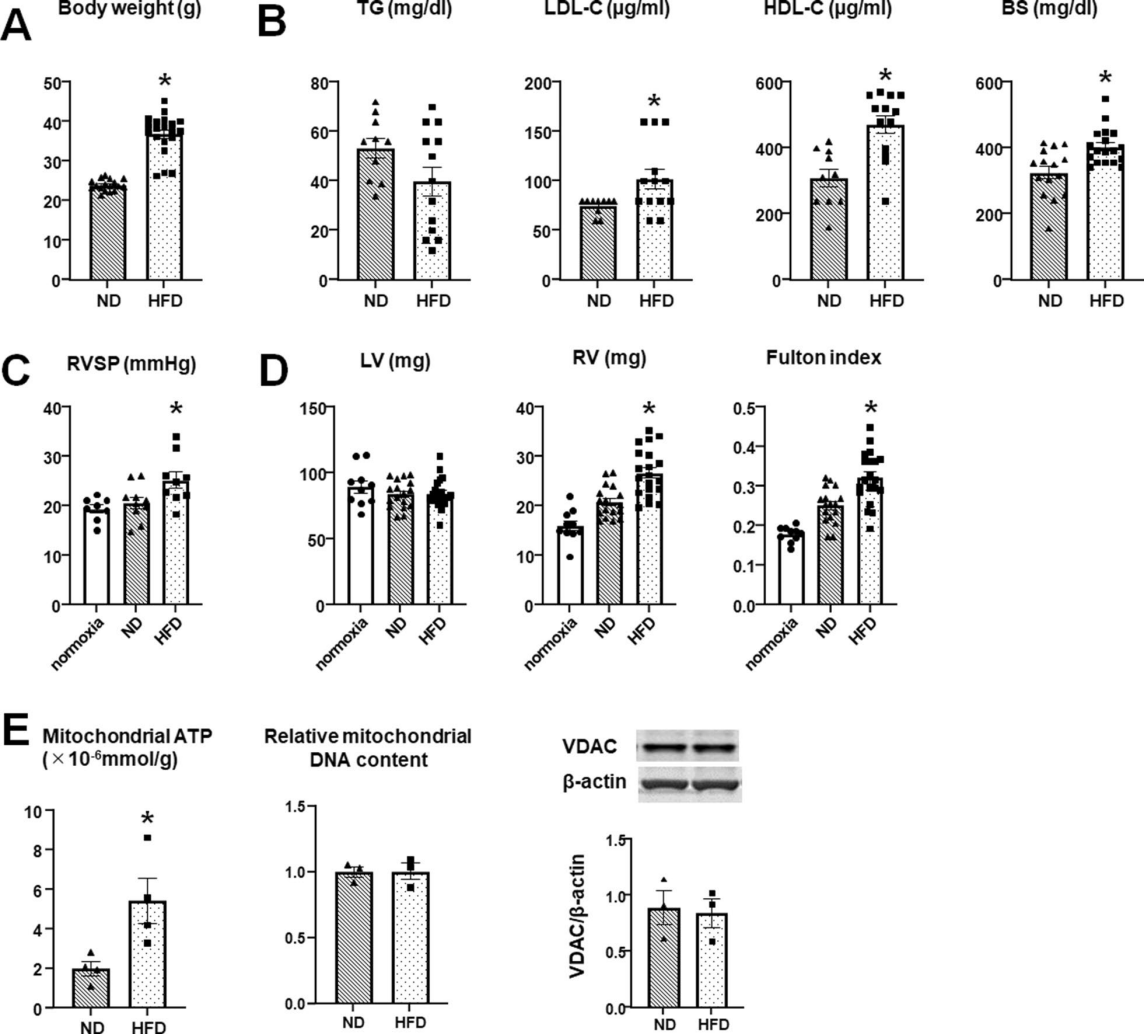
Effect of HFD on reoxygenated hypoxia-induced PH mice. **A** Body weight (ND, n = 17; HFD, n = 20). **B** Levels of TG, LDL-C, HDL-C (ND, n = 10; HFD, n = 13), and glucose (ND, n = 15, HFD, n = 18). **C** RVSP measured by a micromanometer catheter (normoxia, n = 8; ND, n = 10; HFD, n = 9). **D** Weight of the LV and RV, and Fulton index (normoxia, n = 10; ND, n = 17; HFD, n = 20). **E** Levels of mitochondrial ATP, mitochondrial DNA, and VDAC expression (n = 3–4 in each group). The results are expressed as mean ± SEM. *P < 0.05 versus ND group. *ND* normal diet, *HFD* high-fat diet

Figure 3A shows that the body weight in the HFD group is significantly heavier than that in the ND group at 24 weeks in Experiment 2. Glucose, LDL-C, and HDL-C, but not TG, in the HFD group are significantly higher than those in the ND group (Fig. 3B). There is a significant difference in the RVSP of the ND and HFD groups at 24 weeks; that in the ND group almost recovered to normal level, whereas that in the HFD group did not (Fig. 3C). The RV weight and Fulton index in the HFD group are larger than those in the ND group, whereas the LV weight is comparable between the groups (Fig. 3D). Figure 3E shows the mitochondrial ATP levels in the lung tissues. The mitochondrial ATP in the HFD group is higher than that in the ND group. However, mitochondrial DNA and amounts of mitochondrial mass reflected by VDAC expression were equivalent between the groups (Fig. 3E).

### HFD delays reverse remodeling of pulmonary arteries

**Fig. 4:**
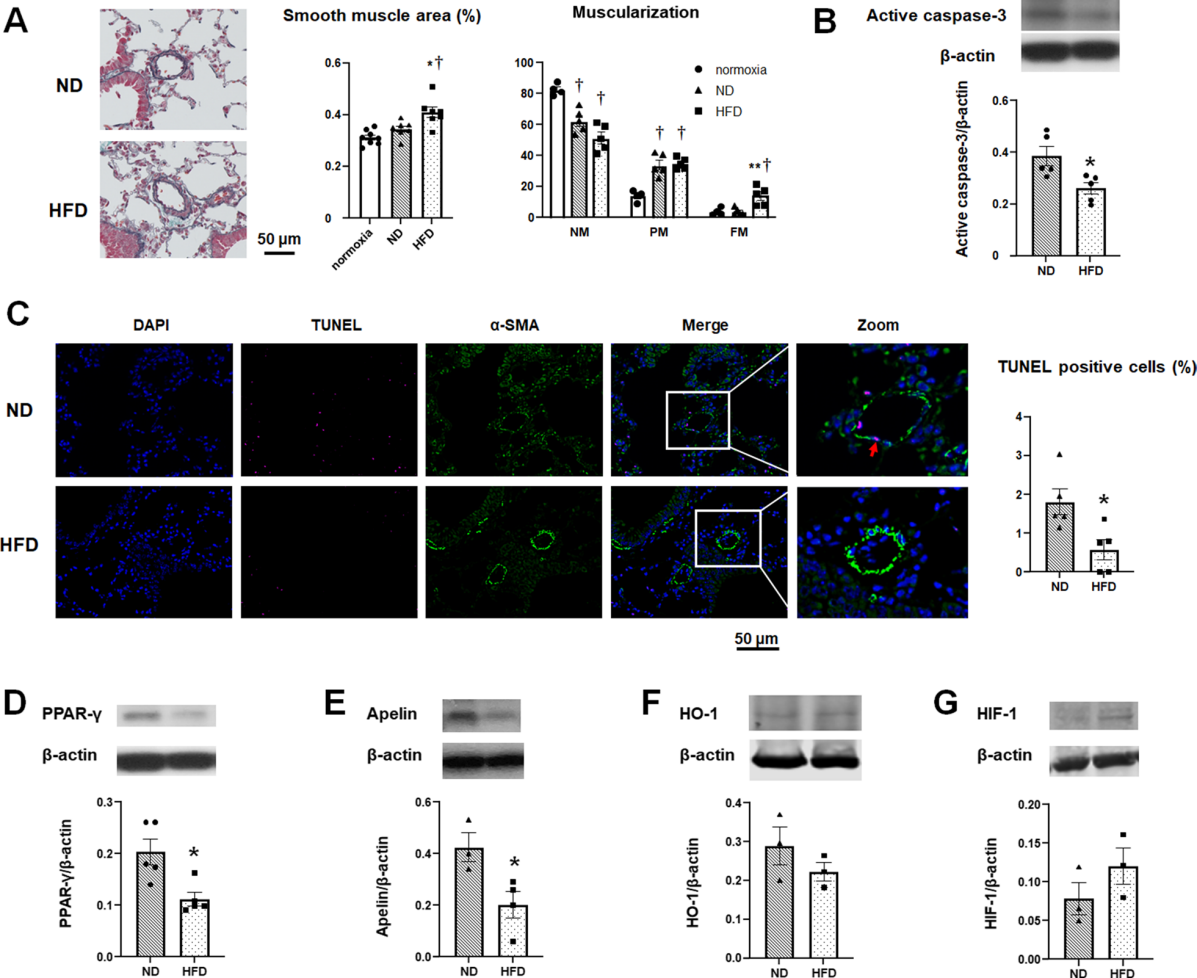
Effect of HFD on pulmonary artery remodeling. **A** Representative microphotographs of pulmonary arteries after EM staining. The smooth muscle area was calculated (n = 7 in each group). After immunostaining with α-SMA, the peripheral pulmonary arteries were classified into non-muscular (NM), partially muscular (PM), and fully muscular (FM) based on the degree of muscularization. The results are expressed as mean ± SEM in 4–5 animals. **B** Active caspase-3 expression in the lung tissues was determined by western blotting. Representative immunoblots (top panels) and bar graph (bottom panel) are shown (n = 5 in each group). **C** Representative microphotographs of TUNEL-stained pulmonary arteries of mice from the ND and HFD group. TUNEL-positive cells are indicated by a red arrow. The graph represents  the percentage of TUNEL-positive nuclei in the medial smooth muscle layer (n = 5 in each group). **D** PPAR-γ expression in the lung tissues was determined by western blotting. Representative immunoblots (top panels) and bar graph (bottom panel) are shown (n = 5 in each group). **E** Apelin expression in the lung tissues was determined by western blotting. Representative immunoblots (top panels) and bar graph (bottom panel) are shown (ND, n = 3; HFD, n = 4). **F**, **G** The expressions of HO-1 **(F)** and HIF-1 **(G)** in the lung tissues were determined by western blotting. Representative immunoblots (top panels) and bar graphs (bottom panel) are shown (n = 3 in each group). The results are expressed as mean ± SEM. *P < 0.05 versus ND group, **P < 0.01 versus ND group, †P < 0.01 versus normoxia-exposed mice. *ND* normal diet, *HFD* high-fat diet

In the ND group, the hypoxia-induced medial wall thickening of the pulmonary arteries almost normalized at 24 weeks, whereas it remained significantly high in the HFD group (Fig. 4A, left and middle panels). The proportion of fully muscular vessels in the HFD group is significantly higher than that in the ND group. The percentages of partially muscular vessels are nearly equivalent between the two groups (Fig. 4A, right panel). Western blotting demonstrated that caspase-3 activity is decreased in the lung tissue of the HFD group (Fig. 4B). TUNEL staining shows that pulmonary artery smooth muscle cell apoptosis in the HFD group is lower than that in the ND group (Fig. 4C). Moreover, the levels of PPAR-γ and apelin are lower in the lung tissue of the HFD group (Fig. 4D, E). There are no significant differences in the levels of HO-1 between the ND group and HFD group (Fig. 4F). HIF-1α is increased in the HFD group, but it is not statistically significant (Fig. 4G).

## Discussion

In this study, HFD suppressed pulmonary artery reverse remodeling and RVSP and RVH improvements in hypoxia-induced PH mice during reoxygenation. One of the possible mechanisms is the undiminished anti-apoptotic pulmonary smooth muscle cells and reduced PPAR-γ and apelin levels in the HFD group. Although there have been several studies reporting that metabolic disorders are associated with the onset and progression of PH in experimental models [19–21], this study is the first to show that HFD affects the improvement process of PH after reoxygenation.

Recently, Umar et al. reported that a western diet increased the lung inflammation, leading to the development of PH in LDL receptor-knockout mice [22]. In this study, although inflammatory changes were not examined, TUNEL-positive cells in pulmonary artery smooth muscle cells and lung caspase-3 activity were more decreased in the HFD group than in the ND group. Since the role of native or oxidized LDL for smooth muscle cell apoptosis or proliferation is controversial [22–26], further studies are needed.

In this study, the HFD group presented higher RVSP and HDL-C levels than the ND group; however, previous studies have shown an association of higher plasma HDL-C levels with good prognosis in human PH [27, 28]. However, the detailed underlying mechanisms responsible for the beneficial effects of HDL-C in pulmonary circulation are not fully explored [28], and a multicenter prospective cohort study by Cracowski et al. showed that HDL-C was not associated with survival from PH [29]. Thus, the role of HDL-C level in PH remains unclear.

Furthermore, Umar et al. showed that a western diet affects left ventricular systolic pressure and RVSP [22]. However, in this study, HFD did not affect the least left ventricular weight.

Chen et al. revealed that mitochondrial dysfunction represented by decreased lung ATP production induces hydrogen peroxide generation and is necessary for smooth muscle cell apoptosis in reverse remodeling during reoxygenation in hypoxia-induced PH mice [4]. This study showed that mitochondrial ATP production was higher in the HFD group than in the ND group. This suggests the possibility that the improvement in mitochondrial function by HFD may have acted suppressively on reverse remodeling. Although HIF-1α is known to inhibit ATP production, there was no significant difference in HIF-1α expression between the ND group and HFD group. In addition, HO-1, as a marker of oxidative stress, was also comparable in spite of the different mitochondrial ATP level. Further mechanisms are needed to be elucidated for these results.

PH patients are reported to have lower levels of plasma apelin, and the administration of apelin agonist improved the hemodynamics in PH patients [11, 30, 31]. A G-protein-coupled receptor, APJ, and its ligand, apelin, are highly expressed in the pulmonary vasculature. It has been reported that apelin-deficient mice develop more severe PH than the wild-type when exposed to hypoxia. Apelin signals are involved in the activation of AMP-activated kinase, Kruppel-like factor 2, and endothelial nitric oxide synthase, and their reduction is thought to reduce nitric oxide-dependent vasodilatation and exacerbate PH [30, 32]. Apelin is regulated by PPAR-γ [11]; thus, it is suggested that the decreased PPAR-γ in reoxygenated HFD mice causes the downregulation of apelin and delays pulmonary vasculature reverse remodeling.

In this study, the ratios of monounsaturated fatty acids/polyunsaturated fatty acids (PUFA) and n-6/n-3 in the diet used in the HFD group were significantly higher than those in the ND group. Previous reports have shown that diets richer in n-3 PUFA suppress pulmonary arterial wall thickening in rats [33]. In addition, the n-3 PUFA enhanced the dilatation of pulmonary vessels through decreased thromboxane A_2_, prostaglandin E_2_, leukotriene B_4_, and interleukin 6, and increased thromboxane A_3_, prostaglandin I_2_, and leukotriene B_5_ [34]. Conversely, n-6 PUFAs have proinflammatory effects. Collectively, it is speculated that the relative increase in n-6/n-3 PUFA in this study may have led to decreased pulmonary vasodilatory effects and increased inflammation, leading to delayed reverse remodeling of the pulmonary arteries.

Until now, the effectiveness of lipid-lowering therapy for pulmonary arterial hypertension (PAH) in previous studies has been controversial despite the association between metabolic disorder and PH [35–37]. In fact, it is confirmed that an intervention for dyslipidemia is less effective than the use of pulmonary vasodilators. However, even though pulmonary vasodilators dramatically improve the prognosis of PAH patients [38], there are cases in which these drugs cannot be used due to side effects. On the other hand, in patients with PH associated with chronic lung disease, pulmonary vasodilator sometimes induces hypoxia due to ventilation/perfusion mismatch. Therefore, it is necessary to establish a treatment other than vasodilator therapy. In addition, we believe that with the improvement in the prognosis of PH patients, the complications of metabolic disorders increase. The results suggest that the treatment for metabolic disorder, in addition to pulmonary vasodilator, has a supportive effect to improve PH.

## Conclusions

In conclusion, this study reports for the first time that a HFD delays the reverse remodeling of the pulmonary arteries of mice with hypoxia-induced PH. Metabolic disorder in PH patients may require active treatment to improve pulmonary artery pressure as soon as possible.

## Supplementary Information

**Additional file 1.** The ARRIVE guidelines 2.0: author checklist.

## Data Availability

All data generated or analyzed during this study are included in this published article.

## Abbreviations

PH: Pulmonary hypertension
PPAR-γ: Peroxisome proliferator-activated receptor-γ
HFD: High-fat diet
RVSP: Right ventricular systolic pressure
RVH: Right ventricular hypertrophy
ND: Normal diet
RV: Right ventricle
LV: Left ventricle
S: Septum
EM: Elastica-Masson
α-SMA: α-Smooth muscle actin
HDL-C: High-density lipoprotein cholesterol
LDL-C: Low-density lipoprotein cholesterol
TG: Triglyceride
ATP: Adenosine triphosphate
TUNEL: Terminal deoxynucleotidyl-transferase-mediated dUTP nick end-labeling
PCR: Polymerase chain reaction
HO-1: Heme oxygenase-1
VDAC: Voltage-dependent anion channel
HIF-1α: Hypoxia inducible factor-1α
